# Distribution of new HIV infections among key risk population groups in Togo

**DOI:** 10.11604/pamj.2014.19.341.4117

**Published:** 2014-12-01

**Authors:** Dadja Essoya Landoh, Angèle Akouavi Maboudou, Kodzo Deku, Palokinam Vincent Pitche

**Affiliations:** 1 Division de l’épidémiologie, Ministère de la Santé, Lomé, Togo; 2 Bureau pays ONUSIDA, Lomé, Togo; 3 Conseil National de lutte contre le SIDA et les IST, Lomé, Togo; 4 Service de Dermatologie et IST, CHU Olympio, Université de Lomé, Togo

**Keywords:** HIV, epidemiology, modes of transmission, Togo

## Abstract

**Introduction:**

Good data on the epidemiology of modes of transmission of HIV among population at risk are important for development of prevention strategies, and resource allocation for the implementation of the interventions. We sought to estimate new HIV infections among key risk groups in Togo.

**Methods:**

We conducted a systematic review of epidemiological data on HIV and AIDS as part of the HIV control strategies in Togo from 2001 to 2012 following the PRISMA guidelines. We used the Mode of Transmission (MoT) modelling tool to estimate the incidence of new HIV infections in high risk groups. The MoT tool was developed and validated by UNAIDS and implemented by several countries using data on the HIV epidemic to estimate new HIV infections that will appear in the core groups. We used Epi-MoT tool to assess the availability and the quality of data. A score of availability of data over 50% and the quality over 1.5 were required to proceed to the MoT analysis. Uncertainty analysis to assess the reliability of the results was performed.

**Results:**

Incidence of new HIV infections was estimated at 6,643 (95% CI = 5274, 9005) with an incidence rate of 203 per 1,000,000 inhabitants. The proportion of new HIV infections was 61.9% (95% CI = 46.2 to 71.7) in stable heterosexual couples compare to 14.01% (95% CI = 7.2 to 23.3) in people having casual sex. In high-risk groups new HIV infections accounted for 2.4% among sex workers (SWs) (95% CI = 1.2 - 4.1), 7.9% among clients of SWs (95% CI = 3.9-14.1) and 6.9% among men who have sex with men (MSM) (95% CI = 3.1 to 13.1).

**Conclusion:**

We describe the prediction of the HIV epidemic with a large contribution of stable heterosexual couples in the occurrence of new infections. But HIV incidence remains high in key risk populations. Innovative strategies for risk reduction should be strengthened to reduce the transmission especially in stable heterosexual couples.

## Introduction

At the end of 2011, UNAIDS estimated the number of people living with HIV/AIDS (PLWHA) to be was 34 million in the world, of which 69% were in living sub-Saharan Africa [1]. It was estimated 2.5 million new infections occurred in 2011 with a decrease of 20% compared to 2001 [1]. Significant progresses have been made in the response to HIV in recent years with improved geographic coverage of different control strategies [2]. In recent years, the financial resources to invest in the control of HIV infection are being scarce [3, 4]. Effective prevention strategies of HIV infection control and resources allocation in a country require knowledge of the epidemiology of transmission modes and the types of higher risk behaviors among key population groups [5]. Know your HIV epidemic package used for analysis and estimation of expected new infections in a population developed is a tool by UNAIDS to help countries assess the proportion of new infections that can occur in the key target populations using epidemiological and socio-behavioral data [5]. This model was therefore recommended to countries in the framework of the initiative “know your epidemic, know your response” to encourage the use of strategic information to help planning of more appropriate and effective responses strategies to the HIV epidemic [5–8]. Togo, with an HIV prevalence of 3.4% in 2011, got the third highest prevalence in West Africa [9]. Since 2001, resources were mobilized for HIV infection control in Togo [10, 11]. Several sero prevalence and behavioral studies, as well as evaluation of the implementation of control strategies have been carried out in the country to better understand the trends of the national HIV epidemic [9]. Mathematical modelling and epidemiological predictions are increasingly used for the development of health policies, the design of prevention programs and allocation of resources for the HIV infection control worldwide [12, 13]. But since the provision of modes of transmission analysis tool, any study has been conducted to estimate new HIV infections by exposure group in Togo. Our objectives were to estimate new HIV infections in the key exposure groups in Togo.

## Methods

We estimate the incidence of new HIV infections among core risk groups using the Know your HIV epidemic package. The modes of transmission (MoT) spreadsheet model was developed and validated by UNAIDS and serves to perform modelling from relevant HIV epidemic data in the country and to determine new infections and major groups among which these new HIV infections may appear [5, 12, 14].

### Study populations

Populations groups at risk defined by the Council of HIV and Control in Togo include female sex workers (SWs) and their clients, injecting drug users (IDUs), men who have sex with men (MSM), people with multiple heterosexual partners, partners of these individuals with higher risk behaviour, people with stable heterosexual relationships mutually faithful (including married and unmarried) and persons who are at risk of infection through medical injections or blood transfusions [5]. In this model, IDUs, MSM and SWs were considered as high-risk groups to HIV while people who have had more than one sexual partner in the last year were considered as people who have casual sex [6, 15].

### Searching strategy

We conducted a systematic review of epidemiological data on HIV and AIDS as part of the HIV control strategies in Togo following the PRISMA guide lines [16]. Recommendations of Meta-analysis of Observational Studies in Epidemiology (MOOSE) statement were also used for conducting and reporting this systematic review [17]. Two investigators (LDE, PVP) helped by a librarian performed a systematic literature search for peer-reviewed studies published between 2001 and 2012 from the following electronic databases: Pub Med, Google scholar and the Scientific Research Journals of Lomé University. We combined a range of the following terms in this review: “HIV prevalence” or Sexual transmitted infection", “MSM” or “Gay”, “Partners of MSM”, “FSW”, “client of FSWs”, “Partners of FSWs’; clients” “IDU” or “Drug users”, “Partners of IDU”, “Casual sex”, “Partners of Casual sex”, “Stable heterosexual”, “Togo”. The literature review was completed by a manual search in the non-computerized institutions using the same targeted terms. We also contacted experts in the field for additional information on unpublished data on HIV and AIDS. All the resulting citations were screened to find out whether they meet the Epi-MoT tool criteria [14, 18].

### Studies eligibility

**Figure 1:**
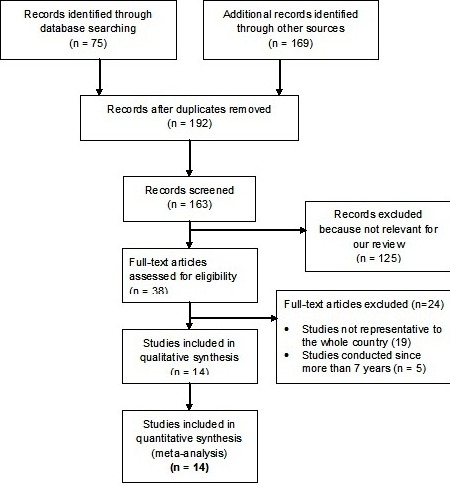
Flow chart of identification and selection of studies for inclusion

For the Epi-MoT requirement, among each risk group, the most recent studies that were conducted in Togo at the national level or are representative to the entire country were eligible [9, 18]. We excluded studies that were not conducted in Togo. Studies that were conducted at district or regional level were excluded. We have summarised the search and selection process for this review in Figure 1.

### Data extraction

For data extraction we used the MoT spreadsheet model requirement described elsewhere [19]. Disagreements on the prevalence of HIV among injecting drug users were resolved by discussion and consensus among reviewers. The model utilises data on the sizes of risk populations, current HIV prevalence, patterns of risk behaviour and levels of protection against HIV infection (reported use of condoms during sexual contact), together with the transmission probabilities associated with specific risk behaviours. It uses a binomial function to estimate the probability of transmission in each identified risk population. The number of sexual/injecting partners and unprotected sex/injecting acts per partner per year determines the annual number of potential risk encounters in each population group, while the likelihood that any contact will be with an HIV infected partner is determined by the HIV prevalence in the corresponding partner population [5] These characteristics from eligible studies were extracted directly into the Epi-MoT tool which is a structured Microsoft Excel spreadsheet called “know your HIV epidemic evaluation package”[5, 19]. Variables recorded included: type of risk group, number of studies reported, year of publication, estimate, geographic area, sample size, population size, HIV prevalence, STI prevalence, sexual behaviour, condom use and number or percent of each risk group receiving ART.

### Data availability and data quality assessment

The Epi-MoT tool (know your HIV epidemic evaluation package) was used to assess the availability of data and the quality of these data [5, 12, 14, 20]. First the epidemiology review checklist was filled out. Then data quality was assessed by giving a quality score ranging from “0” when there is no data available for specific groups: “1” indicating poor data quality, “2” indicating fair and “3” indicating good data quality. The total score of data availability for each population at risk was used as a measure of decision making to proceed to the MoT analysis. To perform MoT modelling and uncertainty analysis, it is recommended that the overall score of the data availability to be greater than 50% and the average of data quality score greater than 1.5 [18]. After validating the data availability, the MoT tool was used to perform the modelling and estimate proportion of new HIV infections by risk group.

### Type of data included in the MoT model

The MoT model uses data on the size of risk population groups, the most recent prevalence of HIV in these risk population groups, the prevalence of sexually transmitted infections as well as the reported condom use during intercostals sex in each population group [5, 18]. The current coverage of antiretroviral therapy by risk group was also introduced [5] in the model. The circumcision rate in Togo was estimated at 98%. The incidence of HIV was estimated between 5,200 and 12,000 new HIV infections at the national level from the spectrum conducted in 2012 [10] and HIV sero prevalence from sentinel surveillance data [9]. The estimate of the size of risk population groups in Togo (Table 1) was made from different studies conducted in Togo and in the West African sub-region [7, 9]. From the data of the general census of population and housing, the size of the population 15 to 49 years in Togo was estimated to be 3,269,420 in 2013 [21].

**Table 1:** Estimation of size of high risk population groups in Togo

Population group	Proportion of population with risk behaviour		Size of population with risk behaviour
	Male	Female	
Injecting Drugs Users (IDU)	0.10	0.00	1637
Partners of IDU	0.00	0.04	638
Sex workers (SWs)	0.00	0.59	9645
Clients des PS	6.11	0.00	99861
Partners of SWs Clients	0.00	0.52	8488
Men having Sex with Men (MSM)	1.00	-	16347
Female partner of MSM	0.00	0.15	2387
Casual sex	19.00	3.80	372714
Partners of casual sex	0.14	3.61	61374
Stable heterosexual couples	73.00	84.00	2566495
No risk	0.65	7.30	129836
Medical Injections	100	100.0	3269420
Blood transfusion	100	100.0	3269420
Total 15 – 49 years population	100	100	3269420

### Analysis

We used the MoT analysis model to estimate the number of new infections expected in a particular population group based on the number of people belonging to the group and the annual risk of infection [5, 12, 14]. The annual risk of infection was determined from the HIV prevalence among each population group, the number of partners in a year, the number of sex contacts per partner per year, the prevalence of other sexually transmitted infections (STIs) in the risk groups and their partners (for sexual transmission only), the proportion of protected sex and the proportion of PLWHA on ART. The model allows to conduct an uncertainty analysis to assess the reliability with 95% confidence interval around our estimates calculated using the uncertainty package incorporated in the MoT model tool [5, 22].

### Ethical aspects

It was a national study commissioned by the National Council for the HIV, AIDS and sexually transmitted infections control of Togo (Ref N° 114/SP/CNLS-IST/CN/2013). The study was conducted using secondary data already available or published. Participants were not involved in this study; therefore, the consents and ethics committee approval were not required.

## Results

Our search yielded 244 records. Of these, 52 duplicate records were excluded. Based on their title and abstracts 125 studies were removed because they were not relevant to the Epi-MoT review. In total, 38 studies were found to be potentially relevant and were assessed. Of these, 24 were excluded because they were not representative to the whole country (n= 18) or they were conducted since more than 7 years (n=6). A total of 14 studies were included in this review (Table 2).

**Table 2:** Characteristics of included studies

Authors and year	Method used	Targeted risk group for HIV reported in the study
Sobela F et al. 2005	Cross sectional Study	Female Sex workers (FSW), Clients of FSWs, Partners of FSW clients
Conseil National de lutte contre le SIDA (CNLS) 2012	Annual report	Blood transfusion
Programme National de Lutte contre le SIDA (PNLS - Togo) 2011	Cross sectional Study	Female Sex workers (FSW), Clients of FSWs, Partners of FSW clients
SODJI KD et al. 2009	Cross sectional Study	Female Sex workers (FSW), Clients of FSWs, Partners of FSW clients
Organisation du Corridor Abidjan Lagos (OCAL) 2012	Cross sectional Study	Men who have Sex with Men (MSM), Injecting Drug users (IDU),
(PNLS - Togo 2010	Cross sectional Study	Men who have Sex with Men (MSM), Partners of MSM
PNLS - Togo 2011	Cross sectional Study	IDU, Partners of IDU
PNLS - Togo 2012	Annual report	Partners of IDU, Partners of MSM, Medical injection, Stable heterosexual, Partners of casual sex, Partners of FSW clients
Unité de Recherche Démographique (URD). 2010	Cross sectional Study	Partners of IDU, Partners of MSM, Casual sex, Stable heterosexual, Partners of casual sex, Partners of FSW clients, Clients of FSW
Population service International (PSI), 2009	Annual report	MSM
PSI, 2010	Cross sectional Study	MSM, Partners of MSM
Direction Générale de la Statistique et de la Comptabilité Nationale 2010	Population survey	Partners of FSW clients, Partners of casual sex, Stable heterosexual, Partners of MSM, Partners of IDU
Conseil National de Lutte contre le SIDA (CNLS) 2012	Annual report	Partners of FSW clients, Partners of casual sex, Stable heterosexual, Partners of MSM, Partners of IDU
PNLS - Togo 2011	Cross sectional Study	Casual sex, Partners of FSW clients

**Availability of data:** the average score of data availability was 68.7% with a highest score of data availability in the SWs population group (Table 3).

**Table 3:** Average score of data availability

Indicators	Average score
Injecting Drug Users	62.5
MSM	62.5
Sex Workers	87.5
Clients of Sex workers	75.0
Casual sex	62.5
Stable heterosexual couples	62.5
No risk	62.5
Average score	68.7

**Figure 2:**
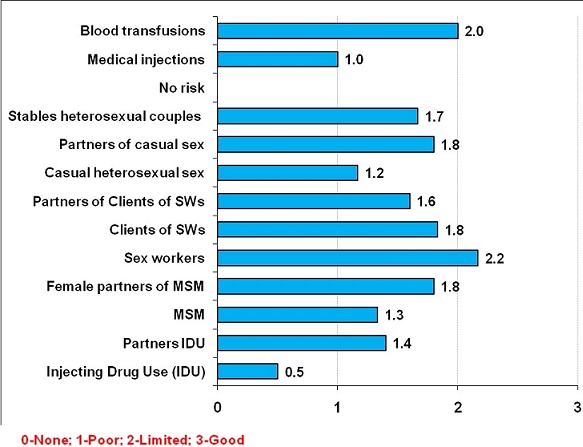
Quality of data available by key risk population group

**Data quality:** The quality of data by key risk groups varied between 0 and 2.2 with an average score of 1.53/3 (Figure 2).

**Characteristics of risk populations:** the size of the population aged 15 to 49 years was estimated at 3,269,420 inhabitants accounting for 48% of the total population of Togo in 2013. Of the 3,269,420 inhabitants, stable heterosexual couples represented 78% and people having casual sex represented 11.4% (Table 4). The size of high risk group population among the population of 15 to 49 years old retrieved from the available studies is shown in Table 1. HIV prevalence was respectively 19.6% among MSM, 13.1% in SWs and 3.1% among clients of SWs (Table 4). The proportion of protected sex acts was 79% in SWs, 72% among MSM, and 61% among clients of SWs (Table 4). Partners of population groups at high risk and stable heterosexual couples had low HIV prevalence however these groups accounted for a large proportion of the sexually active population (Table 4).

**Table 4:** Characteristics of risk population groups

	VIH prevalence (%)	Number of PLWHA*	STI** prevalence (%)	Number of partners per year	Number of exposed acts per partner per year	Sex act protected (%)	PLWHA on ART*** (%)	HIV Incidence	% Incidence 95% CI	Incidence per 100,000
Injecting Drug users (IDU)	5.5	1	6.7	2	224	59.7	0	100	1.50 (0.39 – 2.69)	6098
Partners of IDU	3.4	22	-	2	108	2.5	24.0	8	0.12 (0.05 – 0.08)	1232
Sex workers (SWs)	13.1	1263	36.3	171	9	79.0	15.0	158	2.37 (1.17 – 4.17)	1634
Clients of SWs	5.4	5392	6.8	15	9	61.0	10.0	478	7.20 (3.99 – 14.02)	479
Partners of SWs Clients	3.1	263	-	1	108	2.5	30.0	47	0.71 (0.60 – 0.92)	556
MSM	19.6	3204	27.0	4	10	72.0	10.0	458	6.90 (3.12 – 13.04)	2804
Female partners of MSM	3.1	74	-	2	108	2.5	42.0	136	2.04 (1.15 – 2.41)	5685
Casual sex	4.0	14909	5.0	3	108	57.0	20.0	931	14.01 (7.21 – 23.24)	250
Partners of casual sex	3.1	1903	-	1	108	2.5	30.0	214	3.22 (2.71 – 4.14)	349
Stable heterosexual couples	3.1	79561	3.0	1	108	2.5	35.3	4114	61.92 (46.9 – 71.71)	160
No risk	0.0	1	0.0	0	0	0.0	24.0	0	0 (0,0 - 0,0)	0
Medical Injections	3.1	0	-	1	1	100	-	0	0 (0,0 - 0,0)	0
Blood transfusion	3.1	0	-	1	1	100	-	0	0 (0,0 - 0,0)	0
Total population	3.3	106593	-	-	-	-	30.8	6643	100	203

* PLWHA: People living with HIV/AIDS

** STI: Sexual Transmitted Infections

*** ART: Anti retro Viral Treatment

**Figure 3:**
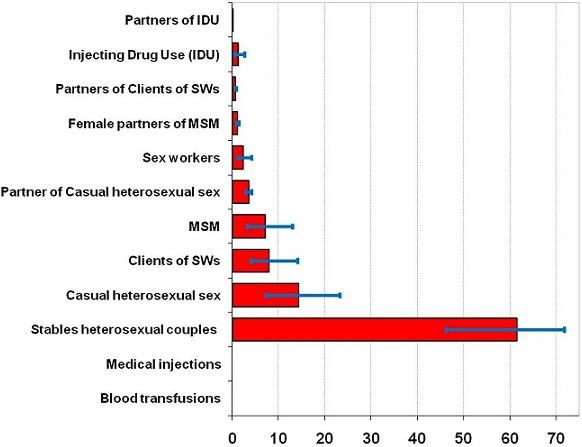
Distribution of new infection by population group

**Distribution of new HIV infections:** the incidence of HIV in Togo was estimated at 6643 new HIV infections in 2013, 95% CI: (5,274 to 9,005). The incidence rate of HIV was 203 cases per 1 million inhabitants. The proportion of the incidence of new HIV infections was 61.9% (95% CI = 46.2 to 71.7) in stable heterosexual couples compare to 14.01% (95% CI = 7.2 to 23.3) in people who have casual sex (Table 4). We found a small percentage of the incidence of new HIV infections among groups with high-risk sexual behaviors such as SWs 2.4%, (95% CI = 1.2 to 4.1), clients of SWs 7.9% (95% CI = 3.9 to 14.1) and MSM 6.9% (95% CI = 3.1 to 13.1) (Figure 3). New HIV infections occur mostly in stable couples 77%, followed by those who have casual sex 17% and partners of high-risk groups (6%).

## Discussion

This study aimed to document the distribution of HIV new infection among key risk group in Togo to better direct resources for effective control of the HIV infection. Hence, it was necessary to conduct a comprehensive review of available HIV [5] epidemiological data. This data analysis was made possible by the Epi-MoT tool that has been developed and used by many countries around the world and especially those in sub-Saharan Africa and Asia [5].

### Availability and quality of data

In the data assessment using Epi-MoT, data availability was 1.53 / 3 while the quality of the data was 68.7% which are acceptable levels for the implementation of the MoT according to the standards recommended by the UNAIDS [18]. Data availability was good for core groups such as SWs and clients of SWs. In fact for a long time, the SWs were considered as high-risk group driving transmission of sexually transmitted infections and HIV in Togo. Hence, resources were mobilized for the control of these infections among this vulnerable group [10, 11]. Population size of high-risk groups (SWs and clients of SWs) in Togo was within the range of West African sub regional estimates [7] while IDUs and MSM are groups that are not yet well organized and documented due to repression and stigma [23–25]. The limited availability of data on IDU and MSM in Togo has affected the quality of data including estimation of the size, the prevalence of STIs and HIV in these population groups.

### Distribution of new HIV infections

MoT model estimated 6643 new HIV infections in Togo. These estimates are within the ranges of predictions made by the Spectrum, which provides an incidence that varies between 5200 and 12,000 new HIV cases in Togo [10]. The number of new infections appear mostly in stable heterosexual couples (77%) followed by those having casual sex (17%). This could be explained by the high proportion of these groups compared to the proportion of SWs, MSM and IDUs in the general population. With HIV prevalence of 3.4%, Togo has a generalized epidemic type, and the MoT model results of Togo confirms the dynamic of this epidemic dominated by heterosexual transmission [5] with a strong female vulnerability [26]. The epidemic in this population group is probably due to the existence of discordant couples and maintained by ignorance of HIV status of one of the partner who has been infected prior to their union. This high incidence of HIV within stable heterosexual couples and those with casual sex has also been reported in most countries of West Africa and Southern Africa [5] and Malawi [8]. Contrariwise, in the East Asia countries, Latin America and Iran [5, 27] where the HIV epidemic is concentrated, predictions reported a larger number of new infections among IDUs and MSM. This difference could be explained by the fact that use of injection drug is not a widespread practice in most countries of sub-Saharan Africa where the prevalence of injection drug use was estimated at 0.2% in the general population [28]. Also, men who have sex with other men are still stigmatized and frowned in African societies. As consequence, the sizes of these population groups are underestimated in most of sub Saharan Africa countries. This is not the case in Asia, America and Europe where, on the one hand these practices are more widespread and sometimes officially recognized. On the other hand, several studies have been conducted in the Western countries in these population groups providing more reliable estimates of the size of these key groups [29, 30]. The prediction of new HIV infection in Togo confirm that SWs, IDU and MSM populations remain at high risk for HIV and strategies to control HIV epidemic must be strengthened for these groups. It is therefore important to improve the mapping of these risk groups for better planning of their health needs. Like any estimation model tool, the MoT tool has structural limits. In fact it does not take into account Mother to child HIV transmission and behavioral aspects of specific age groups such as young people aged 15-24 years. Also some groups or individuals may have multiple partner groups. This could have affected the results from the model.

## Conclusion

Our study describes the prediction of new HIV infection and shows high contribution of stable heterosexual couples in the occurrence of new HIV infections in Togo. However, the incidence of HIV remains relatively high in populations at high risk. These data must be taken into account by the national policies makers for innovative and effective strategies planning to mitigate the number of new HIV infections. Special emphasis should be put on prevention interventions among stable heterosexual couples and key risk populations. In this perspective, these data are taken into account in the ongoing development of the 2013-2017 national plan of elimination of Mother to child HIV transmission, and the development of a framework for investment in the coming years. Moreover, the implementation of new WHO recommendations 2013 [31] on medical management of HIV (CD4 ≤ 500, and the systematic treatment for discordant couples) and the implementation of the strategy of combined prevention among key populations are opportunities to sustainably reverse the trends of the epidemic in 2020 in Togo.

## References

[1] UNAIDS (2012). Report on the global AIDS epidemic.

[2] Saka B, Landoh DE, Patassi A, d'Almeida S, Singo A, Gessner BD (2013). Loss of HIV-infected patients on potent antiretroviral therapy programs in Togo: risk factors and the fate of these patients. Pan Afr Med J..

[3] UNAIDS Regional Support Team for Asia and the Pacific (2008). Redefining AIDS in Asia. Crafting an effective response. Report of the Commission on AIDS in Asia.

[4] Schwartlander B, Stover J, Hallett T, Atun R, Avila C, Gouws E (2011). Towards an improved investment approach for an effective response to HIV/AIDS. Lancet..

[5] Gouws E, Cuchi P (2012). Focusing the HIV response through estimating the major modes of HIV transmission: a multi-country analysis. Sex Transm Infect..

[6] Mumtaz GR, Kouyoumjian SP, Hilmi N, Zidouh A, El Rhilani H, Alami K (2013). The distribution of new HIV infections by mode of exposure in Morocco. Sex Transm Infect..

[7] ONUSIDA RST/WCA (2010). Nouvelles infections du VIH par mode de transmission en Afrique de l'Ouest: une analyse plurinationale.

[8] Maleta K, Bowie C (2010). Selecting HIV infection prevention interventions in the mature HIV epidemic in Malawi using the mode of transmission model. BMC Health Serv Res..

[9] Conseil National de Lutte contre le Sida et le IST Togo (2013). Synthèses des études menées dans le domaine de VIH/SIDA au Togo de 2001 à 2011.

[10] Conseil National de Lutte contre le Sida et le IST Togo (2012). Rapport de progrès sur la riposte au sida au Togo (GARP 2012).

[11] Conseil National de Lutte contre le Sida et le IST Togo (2012). Evaluation des ressources et dépenses consacrées a la réponse nationale au VIH et au sida au Togo (REDES): 2011 et 2012.

[12] Case KK, Ghys PD, Gouws E, Eaton JW, Borquez A, Stover J (2012). Understanding the modes of transmission model of new HIV infection and its use in prevention planning. Bull World Health Organ..

[13] Garnett GP, Cousens S, Hallett TB, Steketee R, Walker N (2011). Mathematical models in the evaluation of health programmes. Lancet..

[14] UNAIDS (2012). Modelling the expected short-term distribution of new HIV infections by modes of transmission.

[15] Conseil National de Lutte contre le Sida et le IST Togo (2012). Plan Stratégique National de Lutte Contre le Sida et les Infections Sexuellement Transmissibles 2012-20.

[16] Moher D, Liberati A, Tetzlaff J, Altman DG, The PG (2009). Preferred Reporting Items for Systematic Reviews and Meta-Analyses: The PRISMA Statement. PLoS Med..

[17] Stroup DF, Berlin JA, Morton SC, Olkin I, Williamson GD, Rennie D (2000). Meta-analysis of observational studies in epidemiology: a proposal for reporting; Meta-analysis Of Observational Studies in Epidemiology (MOOSE) group. JAMA..

[18] UNAIDS (2012). Epidemiological review related to the Modes of Transmission Analysis (Epi-MoT) draft manuel: UNAIDS.

[19] Gouws E, White PJ, Stover J, Brown T (2006). Short term estimates of adult HIV incidence by mode of transmission: Kenya and Thailand as examples. Sex Transm Infect..

[20] Banque Mondiale (2007). Synthèse sur l’épidémiologie du VIH/sida et de la réponse à la problématique en Afrique de l'ouest: Banque Mondiale.

[21] Direction Générale de la Statistique et de la Comptabilité Nationale (DGSCN) (2010). Recensement Général de la Population 2010.

[22] Stover J, Borquez A (2009). Uncertainty estimation for the Modes of Transmission model. UNAIDS.

[23] Programme National de Lutte contre le Sida et le IST Togo (2011). Enquête comportementale et de séroprévalence du VIH chez les hommes ayant des rapports avec d'autres hommes (HSH) au Togo.

[24] Programme National de Lutte contre le Sida et le IST Togo (2011). Enquête comportementale et de séroprévalence du VIH chez les utilisateurs de drogues au Togo.

[25] Takacs J, Kelly JA, P Tóth T, Mocsonaki L, Amirkhanian YA (2013). Effects of Stigmatization on Gay Men Living with HIV/AIDS in a Central-Eastern European Context: A Qualitative Analysis from Hungary. Sex Res Social Policy..

[26] Saka B, Landoh DE, Kombate K, Mouhari-Toure A, Makawa MS, Patassi A (2012). Evaluation of antiretroviral treatment in a cohort of 1,620 HIV-infected patients in Togo. Med Sante Trop..

[27] Nasirian M, Doroudi F, Gooya MM, Sedaghat A, Haghdoost AA (2012). Modeling of human immunodeficiency virus modes of transmission in iran. J Res Health Sci..

[28] Reid SR (2009). Injection drug use, unsafe medical injections, and HIV in Africa: a systematic review. Harm Reduct J..

[29] Van Houdt R, Bruisten SM, Speksnijder AG, Prins M (2012). Unexpectedly high proportion of drug users and men having sex with men who develop chronic hepatitis B infection. J Hepatol..

[30] Ezoe S, Morooka T, Noda T, Sabin ML, Koike S (2012). Population size estimation of men who have sex with men through the network scale-up method in Japan. PLoS One..

[31] World Health Organization (2013). Consolidated guidelines on the use of antiretroviral drugs for treating and preventing HIV infection.

